# Perception of Person-Centred Maternity Care and Its Associated Factors Among Post-Partum Women: Evidence From a Cross-Sectional Study in Enugu State, Nigeria

**DOI:** 10.3389/ijph.2021.612894

**Published:** 2021-06-17

**Authors:** Daniel C. Ogbuabor, Chikezie Nwankwor

**Affiliations:** ^1^ Department of Health Administration and Management, College of Medicine, University of Nigeria, Enugu, Nigeria; ^2^ Department of Health Systems and Policy, Sustainable Impact Resource Agency, Enugu, Nigeria

**Keywords:** person-centred care, maternity care, respectful maternity care, responsive care, facility-based childbirth, Nigeria

## Abstract

**Objectives:** This study validated a person-centred maternity care (PCMC) scale and assessed perception of PCMC and its associated factors among post-partum women.

**Methods:** A cross-sectional study was conducted among 450 post-partum women in two districts in Enugu State, Nigeria, using a 30-item PCMC scale. Exploratory and confirmatory factor analyses, descriptive, bivariate and Generalized Linear Models analyses were conducted.

**Results:** Twenty-two items were retained in the PCMC scale with high internal reliability and goodness-of-fit indices. About 25% of women received high PCMC. Marrying at 20–29 years (β = 3.46, ρ = 0.017) and 30–49 years (β = −5.56, ρ = 0.020); self-employment (β = −7.50, ρ = 0.005); marrying government worker (β = 7.12, ρ = 0.020); starting antenatal care in the third trimester (β = −6.78, ρ = 0.003); high participation in decision-making (β = −10.41, ρ < 0.001); domestic violence experience (β = 3.60, ρ = 0.007); delivery at health centre (β = 18.10, ρ < 0.001), private/mission hospital (β = 4.01, ρ = 0.003), by non-skilled attendant (β = −16.55, ρ < 0.001) and community health worker (β = −10.30, ρ < 0.001); and pregnancy complication (β = 4.37, ρ = 0.043) influenced PCMC.

**Conclusion:** The PCMC scale is valid and reliable in Nigeria. PCMC requires improvement in Enugu State. This study identified factors that may be considered for inclusion in intervention strategies.

## Introduction

Person-centred maternity care (PCMC), defined as “maternity care that is respectful of and responsive to individual women and their families’ preferences, needs, and values” [1], is an effective strategy for improving quality of care experienced by women during facility-based childbirth in low- and middle-income countries (LMICs) [2, 3]. Improvements in quality of maternity care contribute to a reduction in maternal mortality ratio [2]. PCMC, comprising autonomy and communication, respect and dignity, and supportive care, aims at reducing mistreatment, abuse, disrespect and neglect of women during facility-based childbirth; and promoting positive childbirth experiences [1, 4]. Prior studies indicate that women are not getting adequate PCMC in LMICs [1, 4–6]. Perception of PCMC ranged from low in Ghana to moderately high in Kenya and India [1, 4–6]. Women’s perception of dignity and respect was high; communication and autonomy, low to moderately high; and supportive care, moderately high to high [1, 4–6]. Neglect of PCMC leads to disparities in use of skilled birth attendance and maternal and neonatal outcomes [7].

Broadly, patient characteristics, facility characteristics, and service types affect PCMC [3]. Patient characteristics include socio-demographic characteristics, clinical history and prior health care-seeking behaviour [3]. Perception of PCMC varied with socio-economic status and education in Kenya, India and Ghana [5, 7]. Women with high socio-economic status are usually personally empowered; live in areas with high quality of care; tend to have relationships with healthcare providers, narrow social power between women and providers, higher expectation of care, and capacity to advocate for high quality care [7, 8]. Higher incidents of disrespectful and abusive care were reported from women from lower socio-economic strata in Ethiopia and Pakistan [9–11], and younger and less educated women in other LMICs [12]. Employment, marital status and absence of domestic violence experience predicted higher PCMC among childbearing women [7]. In this study, we hypothesize that low socio-economic status, experience of domestic violence and low participation in household decisions will be associated with low PCMC.

Facility characteristics such as type of facilities and types of providers may modify women’s experiences of maternity care [3]. Whereas indices of clinical quality of maternal health were higher in hospitals than health centres [13, 14], interpersonal quality was higher in private than public hospitals [13, 15]. Women who delivered in health centres and private hospitals reported higher PCMC than those who were delivered in public hospitals in Kenya and India, but no significant differences were observed in Ghana [5, 7]. Disrespect and abuse were more likely in hospitals than health centres in Ethiopia [10], and in public health facilities than private health facilities in Pakistan [9]. In this study, we hypothesize that PCMC will be lower in public hospitals than health centres and private hospitals.

Evidence that type of services influences PCMC are mixed. In Pakistan, type of delivery service did not influence women’s experiences of disrespect and abuse [9]. Pregnancy complications was related to high perception of PCMC in Ghana, but not in Kenya and India [5]. However, severe pregnancy complication significantly predicted higher PCMC in Kenya [7]. Also, In Gambia, normal vaginal delivery was associated with a higher perception of autonomy and supportive care than instrumental delivery [16]. Conversely, higher incidents of disrespectful and abusive care were reported from women who had complications, longer labour durations and Caesarean birth [11, 17–19]. Our proposition is that women with pregnancy complications will have significantly lower PCMC than those without complications.

In Nigeria, mistreatment of women during childbirth are common and not only undermine utilization of health facilities for delivery but also create psychological distance between women and health providers [20, 21]. Yet, respectful and responsive maternity care has not been comprehensively studied in Nigerian health system [20, 22]. Also, PCMC scale has not been validated nor has any study investigated women’s perception and determinants of PCMC in Nigeria. This study, therefore, validated the PCMC scale, assessed perception of PCMC and its associated factors among post-partum women in Enugu State, Nigeria. This evidence will help decision-makers, providers, and service users identify gaps, design interventions to promote positive childbirth experiences, and evaluate changes in quality of maternity care.

## Methods

### Study Setting

The study took place in two districts of Enugu State, South-east Nigeria. Enugu state was delineated into seven health districts. We categorised the seven health districts into two groups of three well-performing and four less-performing districts using maternal healthcare utilisation data [23]. Enugu Metropolis and Isi-Uzo districts were randomly selected from the well-performing and less-performing districts, respectively. The two districts have, each a general hospital and a network of cottage hospitals and primary health facilities. In 2019, the estimated population of Enugu State was about 4.8 million people. Enugu Metropolis and Isi-Uzo had 1,061,256 and 217,952 populations respectively, out of which women of childbearing age constitute 47.2 and 43.1% respectively [24]. Skill birth attendance is about 93% [24]. However, the maternal mortality ratio in Enugu is 1,252/100, 000 live births [25], higher than the national ratio of 512/100,000 live births [24].

### Research Design

The study adopted a facility-based cross-sectional survey design using an interviewer administered questionnaire.

### Study Population and Sampling Strategy

Post-partum women aged 15–49 years, who delivered in 9 weeks preceding the study constituted the study population. To detect mean differences between post-partum women in the two districts (alpha level = 0.05, 95% power, allocation ratio of 3:1, GPower 3.1.9.7), we required a minimum sample size of 280 (70 in Isi-Uzo and 210 in Enugu metropolis). We, however, sampled 450 eligible post-partum women equally allocated to the two districts.

In each district, we purposively selected the general hospital and four primary health centres (one facility per local health authority) with the highest maternal and child healthcare attendance based on routine health management information system. Additionally, the sample in Enugu metropolis purposively included the state teaching hospital because of its central location which made it very accessible. Eligible post-partum women were recruited by convenience as they leave immunisation clinics using healthcare providers as gatekeepers.

### Data Collection

Data was collected from January to March 2019 using an interviewer-administered PCMC scale made up of 30 items measuring three domains of PCMC: dignity and respect (6 items), communication and autonomy (9 items), and supportive care (15 items) [1]. The PCMC scale has been validated in similar low-resource context with good reliability coefficients for the total PCMC scale and sub-scales [1, 4]. The Cronbach alpha coefficients for full PCMC scale, dignity and respect (DR), autonomy and communication (AC), and supportive care (SC) sub-scales in Kenya were 0.86, 0.63, 0.73, and 0.72 correspondingly [1]. In India, the Cronbach alpha coefficients for full PCMC scale (27 items), DR (6 items), AC (9 items), and SC (12 items) sub-scales were 0.85, 0.70, 0.67, and 0.73, respectively [4]. Each item is on a 4-point response scale—0: “no, never,” 1: “yes, a few times,” 2: “yes, most of the time,” and 3: “yes, all the time.” For each respondent, responses from the PCMC scale were summed up into one composite PCMC score. The possible score on the PCMC scale range from 0 to 90, with a lower score implying poorer PCMC. The range of possible scores on the sub-PCMC scales are: 0–18, 0–27, and 0–45 for respect and dignity, communication and autonomy, and supportive care correspondingly.

The questionnaire also included information on socio-demographic characteristics such as age, marital status, residence, religion, age at marriage, education, literacy, occupation, partner’s education, partner’s occupation, and maternal health care-seeking behaviour. Other information collected include facility characteristics (facility type and provider type), service types, household wealth index, women’s participation in household decisions, domestic violence tolerance, and experience as well as a question on overall satisfaction with maternity care. Household wealth index was measured using 11 questions on Nigeria equity tool and its accompanying syntax used to create wealth quintiles [26]. Participation in household decision-making was assessed using questions on five household decisions [27]. Each question was assigned the following scores: 0—if the decision was made by husband/partner alone, someone else or other; 1—if the decision was jointly made by respondent and husband/partner; and 2—if the respondent alone made the decision. Participation score ranged from 0–10. Also, attitudes towards domestic violence were measured using five variables describing whether beating was justified if the wife: goes out without telling her husband; neglects the children; argues with her husband; refuses sex with her husband; and burns food [27]. Women who answered “Yes” and “Don’t know” were scored 0 while women who responded “No” were scored 1. Domestic violence tolerance score ranged from 0–5. The value of either the participation score or domestic violence tolerance was transformed into 0–1 interval [27]. The median values were used to dichotomise the scores into low and high participation as well as domestic violence tolerant and intolerant categories. Five trained research assistants administered the questionnaires, while the authors supervised the data collection.

### Data Analysis

We conducted exploratory and confirmatory factor analyses using EViews version 11 and all other analyses using SPSS (version 26, IBM, NY, United States). Data were assessed for sampling adequacy using Kaiser-Meyer-Olkin measure. We conducted exploratory factor analysis with maximum likelihood estimation. Items that yield communalities ≥0.4 were deemed adequate [28]. A rotated factor loading of 0.32 on Promax rotation with Kaiser Normalization was considered significant [28]. The goodness of fit of the final factor structure of the 22-item scale was assessed using Chi-square goodness of fit test with confirmatory factor analysis. Additionally, a series of goodness-of-fit indices (root mean square residual, generalized fit index, adjusted generalized fit index, root mean square error of approximation, normed fit index, non-normed fit index, incremental fit index, and comparative fit index) were used to evaluate the quality of model fit. Cronbach alpha, inter-item correlation and intraclass correlation were used to report the reliability coefficients of the PCMC scale and sub-scales.

Characteristics of respondents were presented using frequencies and percentages. Mean PCMC scores and standard deviation were calculated and compared across various socio-demographic characteristics of respondents, facility characteristics and service type using t-tests and analysis of variance (ANOVA). Parametric tests were deemed appropriate since the single composite PCMC scores have interval-like properties. We categorized full PCMC and each sub-scale into “low, medium, and high.” Low was defined as scores in the approximate lower 25th percentile and scores in the top 75th percentile defined as high [29]. Pearson correlation was used to test association of total PCMC scores with overall satisfaction with maternity care. Generalized Linear Models was used to test relationship between PCMC and the parameters that were significant on bivariate analysis. Statistical significance was set at alpha 0.05 level.

### Ethical Consideration

The study was approved by the Health Research Ethics Committee of University of Nigeria Teaching Hospital, Enugu, Nigeria (NHREC/05/01/2008B-FWA00002458-IRB00002323). Written, informed consent was obtained from all respondents.

## Results

### Characteristics of Respondent

The response rate was 100%. Table 1 shows the characteristics of respondents. Most women were married, Christians, Igbo, married in their 20 s and educated to at least secondary or vocational school. About a fifth of women were unemployed. While 57% of women had low participation in household decisions, about 30% had experienced domestic violence. About 54% of women started antenatal care late. About 8% reported pregnancy complications.

**TABLE 1:** Characteristics of respondents, Enugu State, Nigeria, 2019.

Parameters		Frequency (n)	Percent (%)
Age	15–19 years	25	5.6
	20–29 years	259	57.6
	30–49 years	166	36.9
Marital status	Married	444	98.7
	Not married	6	1.3
Religion	Christianity	447	99.3
	Muslim	3	0.7
Ethnicity	Igbo	444	98.7
	Others	6	1.3
Residence	Urban	225	50.0
	Rural	225	50.0
Age at marriage	15–19 years	93	20.7
	20–29 years	322	71.5
	30–49 years	35	7.8
Parity	1	134	29.8
	2	130	28.9
	3	103	22.9
	≥4	83	18.4
Educational level of respondent	—	—	—
	No school/primary	55	12.2
	Secondary/Vocational	262	58.2
	Tertiary	133	29.6
Literacy rate writing	No, cannot write	59	13.1
	Yes, with some difficulty	157	34.9
	Yes, very well	234	52.0
Literacy rate reading	No, cannot read	58	12.9
	Yes, with some difficulty	155	34.4
	Yes, very well	237	52.7
Wealth index	Poorest	66	14.7
	Poorer	59	13.1
	Middle	57	12.7
	Richer	68	15.1
	Richest	200	44.4
Occupation of respondent	Unemployed/student	89	19.8
	Self employed	148	32.9
	Salaried worker	56	12.4
	Artisan/petty trading	97	21.6
	Farmer	60	13.3
Employment of respondent	Unemployed	89	19.8
	Employed	361	80.2
Partner’s occupation	Unemployed/student	17	3.8
	Self employed	189	42.0
	Salaried worker	96	21.3
	Artisan/petty trading	99	22.0
	Farmer	49	10.9
Partner’s educational level	—	—	—
	No school/primary	84	18.7
	Secondary/Vocational	226	50.2
	Tertiary	140	31.1
Health insurance	No	412	91.6
	Yes	38	8.4
Participation in decision making	Low participation	258	57.3
	High participation	192	42.7
Domestic violence attitude	DV tolerance	45	10.0
	DV intolerance	405	90.0
Domestic violence experience	No	316	70.2
	Yes	134	29.8
Delivery facility type	Public hospital	160	35.6
	Public health centre	130	28.9
	Private/mission	160	35.6
Provider type	Nurse/midwife	117	26.0
	Doctor	86	19.1
	Community health worker	201	44.7
	Non-skilled attendant	46	10.2
Pregnancy complication	No	413	91.8
	Yes	37	8.2
Previous facility delivery	No	172	38.2
	Yes	278	61.8
Trimester of first ANC	No ANC	5	1.1
	First trimester	200	44.4
	Second trimester	213	47.3
	Third trimester	32	7.1
Number of ANC visits	No ANC	8	1.8
	<4	37	8.2
	4 or 5	161	35.8
	≥6	244	54.2
Postpartum length	1 week	43	9.6
	2 weeks	12	2.7
	3 weeks	5	1.1
	4 weeks	25	5.6
	5–9 weeks	365	81.1

### Factor Analysis

The PCMC scale was found to be valid. The Kaiser-Meyer-Olkin measure of sampling adequacy was 0.9507 (X^2^ = 1886, ρ < 0.001). Eight items yielded communalities ≤0.4, which were deemed inadequate (Table 2). Promax rotation with Kaiser Normalization showed that the remaining 22 items loaded ≥0.32 and were retained. Twenty items loaded on factor 1 (87.8% variance), while only two items loaded on factor 2 (12.2% variance). The goodness of fit Chi-square statistic was 1150.305 (ρ < 0.001). Goodness-of-fit indices suggested adequate fitness as shown in Table 3. PCMC scores correlated strongly with women’s overall satisfaction with quality of maternity care (*r* = 0.910, ρ < 0.001) indicating high criterion validity. The possible score on our 22-item PCMC scale range from 0 to 66. The range of possible scores on the sub-PCMC scales are: 0–12, 0–21, and 0–33 for respect and dignity (*n* = 4), communication and autonomy (*n* = 7), and supportive care (*n* = 11) correspondingly. Full PCMC scores correlated strongly with women’s overall satisfaction with quality of maternity care (*r* = 0.910, ρ < 0.001) indicating high criterion validity. The reliability indices of the full PCMC scale and sub-scales were high as shown in Table 3.

**TABLE 2:** Items deleted from person-centred maternity care scale during factor analysis for different reasons, Enugu State, Nigeria, 2019.

PCMC domain	Item	Theme	Reason for deletion
Dignity and respect	Did the doctors, nurses or other staff shout at you, scold, insult, threaten, or talked to you rudely?	Verbal abuse	Low communality
	Did you feel like you were treated roughly like pushed, beaten, slapped, pinched, physically restrained, or gagged?	Physical abuse	Low communality
Autonomy and communication	During your time in the health facility, did the doctors, nurses, or other healthcare providers introduce themselves to you when they first came to see you?	Introduce self	Low communality
	During your delivery, do you feel like you were able to be in the position of your choice?	Delivery position	Low communality
Supportive care	Did you feel you waited long before the health workers attended to you?	Time to care	Low communality
	Were you allowed to have someone you wanted to stay with you during delivery?	Delivery support	Low communality
	Thinking about the labour and postnatal wards, did you feel the health facility was crowded?	Crowded	Low communality
	Was there clean drinking water available in the facility?	Clean water	Low communality

**TABLE 3:** Reliability and goodness-of-fit indices of person-centred maternity care scale in Enugu State, Nigeria, 2019.

	Full PCMC	Dignity and respect	Autonomy and communication	Supportive care
	**(*n* = 22)**	**(*n* = 4)**	**(*n* = 7)**	**(*n* = 11)**
Reliability indices				
Cronbach alpha	0.94	0.94	0.91	0.82
Intraclass correlation	0.96*	0.94*	0.92*	0.87*
Common inter-item correlation	0.43	0.80	0.59	0.29
Goodness-of-fit indices	—	—	—	—
Chi-square statistic	1,150.305	—	—	—
Chi-square probability	<0.001	—	—	—
Root mean square residual. (RMSR)	0.032	—	—	—
Generalized fit index (GFI)	0.798	—	—	—
Adjusted GFI	0.729	—	—	—
Root mean square error of approximation	0.107	—	—	—
Bentler-bonnet Normed (NFI)	0.893	—	—	—
Tucker-lewis Non-Normed (NNFI)	0.887	—	—	—
Bollen incremental (IFI)	0.909	—	—	—
Bentler comparative (CFI)	0.908	—	—	—

Recommended cut off [28]: ≥0.9 for GFI, AGFI, NFI, NNFI, IFI, CFI; ≤ 0.1 for RMSR; ≤ 0.08 for RMSEA.

*Significant at ρ < 0.001.

### Distribution of Individual PCMC Items Among Women (N = 450)

As shown in Table 4, most women were treated with respect and in a friendly manner Most women also reported good visual privacy and record confidentiality.

**TABLE 4:** Distribution of individual person-centred maternity care items (*n* = 22) among post-partum women in Enugu State, Nigeria, 2019.

PCMC domain	Item	Response	Frequency (n)	Percent (%)
Dignity and respect	Treated with respect	Few times or never	94	20.9
		Most or all the time	356	79.1
	Friendly	Few times or never	30	6.7
		Most or all the time	420	93.3
	Visual privacy	Few times or never	100	22.2
		Most or all the time	350	77.8
	Record confidentiality	Few times or never	100	22.2
		Most or all the time	350	77.8
Autonomy and communication	Called by name	Few times or never	146	32.4
		Most or all the time	304	67.6
	Involvement in care	Few times or never	142	31.6
		Most or all the time	308	68.4
	Consent to procedures	Few times or never	110	24.4
		Most or all the time	340	75.6
	Language	Few times or never	93	20.7
		Most or all the time	357	79.3
	Explain exams/procedure	Few times or never	111	24.7
		Most or all the time	339	75.3
	Explain medicine	Few times or never	111	24.7
		Most or all the time	339	75.3
	Able to ask question	Few times or never	95	21.1
		Most or all the time	355	78.9
Supportive care	Labour support	Few times or never	335	74.4
		Most or all the time	115	25.6
	Talk about feeling	Few times or never	111	24.7
		Most or all the time	339	75.3
	Support anxiety	Few times or never	191	42.4
		Most or all the time	259	57.6
	Attention when need help	Few times or never	90	20.0
		Most or all the time	360	80.0
	Took best care	Few times or never	90	20.0
		Most or all the time	360	80.0
	Control pain	Few times or never	205	45.6
		Most or all the time	245	54.4
	Trust	Few times or never	88	19.6
		Most or all the time	362	80.4
	Enough staff	Few times or never	131	29.1
		Most or all the time	319	70.9
	Cleanliness	Few times or never	103	22.9
		Most or all the time	347	77.1
	Electricity	Few times or never	104	23.1
		Most or all the time	346	76.9
	Safe	Few times or never	90	20.0
		Most or all the time	360	80.0

Most women reported that providers called women by their names, involved women in care decisions, sought consent to procedures, talked to women in language that women understood, explained examinations and medicines (Table 4). Likewise, most women were able to ask their service providers questions.

Most women indicated that providers paid attention when needed, talked to women about their feeling, took the best care of them and could be trusted (Table 4). Most women also reported that health facilities were safe, clean, had electricity but had few staff. A quarter of women indicated that health facilities did not allow labour support (Table 4).

### Distribution of Full PCMC Scale and Sub-Scales

Overall, women had medium scores on full PCMC scale and sub-scales, but a quarter of women perceived PCMC as high (Table 5). Over 60% of women perceived respect and dignity as high. While less than a quarter of women perceived communication and autonomy as high, just over a quarter perceived supportive care as high.

**TABLE 5:** Descriptive statistics of person-centred maternity care scale and sub-scales in Enugu State, Nigeria, 2019.

Outcome	Frequency (n)	Percent (%)	Mean (SD)	Percentiles	
				25^th^	75^th^
Full PCMC (*n* = 22)	N = 450		49.07 (15.41)	48.00	60.00
Low	107	23.8			
Medium	229	50.9			
High	114	25.3			
Dignity and respect sub-scale (*n* = 6)	N = 450		9.95 (3.20)	9.00	12.00
Low	109	24.2			
Medium	56	12.5			
High	285	63.3			
Communication and autonomy sub-scale (*n* = 8)	N = 450		15.87 (5.44)	13.75	20.00
Low	112	24.9			
Medium	199	52.0			
High	139	23.1			
Supportive care sub-scale (*n* = 13)	N = 450		23.25 (7.40)	22.75	28.00
Low	112	24.9			
Medium	217	48.3			
High	121	26.8			

PCMC, Person-centred maternity care; SD, Standard deviation.

### Bivariate Analysis

Table 6 shows mean score differences in PCMC disaggregated by patient characteristics, facility characteristics, and service types. Women who married at age 20–29 years had significantly higher mean PCMC score than women in other age groups (ρ < 0.001). Self-employed women (ρ < 0.001) and women married to unemployed partners (ρ = 0.008) had significantly low PCMC scores among occupational categories. High participation in household decision-making (ρ < 0.001), domestic violence experience (ρ < 0.001) and starting antenatal care during the third trimester (ρ< 0.001) were associated with lower PCMC scores. Delivery in health centres (ρ < 0.001), delivery by CHEW (ρ < 0.001), and pregnancy complications (ρ = 0.030) were associated with higher PCMC scores.

**TABLE 6:** Mean perception of person-centred maternity care disaggregated by respondents’ characteristics in Enugu State, Nigeria, 2019.

Parameters	Age	Mean^a^	Std. Deviation	Sig
Age^b^	15–19 years	48.88	15.89	0.721
	20–29 years	49.56	15.28	—
	30–49 years	48.33	15.62	—
Marital status^c^	Married	49.23	15.33	0.053
	Not married	37.00	18.33	—
Religion^c^	Christianity	49.12	15.42	0.384
	Muslim	41.33	19.40	—
Ethnicity^c^	Igbo	49.09	15.47	0.761
	Others	47.17	10.91	—
Residence^c^	Urban	48.03	14.93	0.153
	Rural	50.11	15.85	—
Age at marriage^c^	15–19 years	39.69	18.04	<0.001*
	20–29 years	51.22	13.79	—
	30–49 years	45.16	17.63	—
Parity^b^	1	49.36	15.57	0.248
	2	50.65	14.50	—
	3	46.57	16.09	—
	≥4	49.22	15.41	—
Educational level of respondent^b^	No/primary school	48.27	16.18	0.857
	Secondary/vocational	49.39	15.28	—
	Tertiary	48.77	15.45	—
Literacy rate writing^b^	No, cannot write	50.95	14.31	0.341
	Yes, with some difficulty	49.80	15.05	—
	Yes, very well	48.10	15.91	—
Literacy rate reading^b^	No, cannot write	50.95	14.46	0.548
	Yes, with some difficulty	49.24	15.49	—
	Yes, very well	48.50	15.61	—
Wealth index^b^	Q1	51.12	15.62	0.483
	Q2	47.04	17.45	—
	Q3	47.15	16.52	—
	Q4	51.29	14.93	—
	Q5	48.63	15.23	—
Occupation of respondent^b^	Unemployed/student	48.46	15.69	<0.001*
	Self-employed	43.96	17.96	—
	Salaried worker (govt.)	51.66	14.24	—
	Artisan/petty trading	54.26	9.25	—
	Farmers	51.77	13.58	—
Employment of respondent^c^	unemployed	48.46	15.69	0.678
	Employed	49.22	15.36	—
Partner’s occupation^b^	Unemployed/student	45.71	17.41	0.008*
	Self-employed	46.48	17.19	—
	Salaried worker (govt.)	49.76	14.86	—
	Artisan/petty trading	53.09	10.78	—
	Farmers	50.76	14.93	—
Partner’s educational level^b^	No/primary school	47.23	16.93	0.390
	Secondary/vocational	49.90	14.73	—
	Tertiary	48.84	15.55	—
Health insurance^c^	No	49.14	15.37	0.737
	Yes	48.26	16.06	—
Participation in decision making^c^	Low	54.68	8.91	<0.001*
	High	41.53	18.76	—
Domestic violence tolerance^c^	DV tolerance	50.56	12.80	0.490
	DV intolerance	48.88	15.70	—
Domestic violence experience^c^	No	52.96	11.09	<0.001*
	Yes	47.42	16.66	—
Delivery facility type^b^	Public hospital	44.24	16.99	<0.001*
	Public health centre	55.50	8.75	—
	Private/mission hospital	48.67	16.21	—
Provider type^b^	Nurse/Midwife	48.46	15.45	<0.001*
	Doctor	50.50	14.31	
	CHEW	51.50	13.97	—
	Non-skilled attendant	26.31	17.61	—
Pregnancy complication^c^	No	48.60	15.73	0.030*
	Yes	54.32	10.12	—
Previous facility delivery^c^	No	49.32	15.58	0.786
	Yes	48.91	15.34	—
Trimester of first ANC^b^	No ANC	40.20	17.27	<0.001*
	First trimester	49.52	15.02	—
	Second trimester	50.61	14.40	—
	Third trimester	37.38	19.07	—
Number of ANC visits^b^	No ANC	47.00	16.12	0.326
	<4	45.27	16.54	—
	4 or 5	50.31	14.22	—
	6+	48.89	15.95	—
Postpartum length^b^	1 week	52.14	11.82	0.504
	2 weeks	51.50	15.97	—
	3 weeks	44.40	13.67	—
	4 weeks	51.16	12.52	—
	>/ = 5 weeks	48.55	15.96	—

a Reported on a scale of 0–81 with higher scores corresponding to higher PCMC.

b ANOVA.

c t-test.

*Significant at ρ ≤ 0.05.

### Factors Influencing Person-Centred Maternity Care

Table 7 shows the parameters that influenced perceived PCMC among women in this study. Marrying at age 20–29 years (β = 3.46, ρ = 0.017) and 30–49 years (β = −5.56, ρ = 0.020), self-employed women (β = −7.50, ρ = 0.005), married to a government worker (β = 7.12, ρ = 0.020), starting antenatal care in the third trimester (β = −6.78, ρ = 0.003), high participation in household decisions (β = −10.41, ρ ≤ 0.001), domestic violence experience (β = 3.60, ρ = 0.007), delivery at health centre (β = 18.10, ρ < 0.001), delivery at private/mission hospital (β = 4.01, ρ = 0.003), delivery by non-skilled attendant (β = −16.55, ρ < 0.001); and delivery by community health worker (β = −10.30, ρ < 0.001) and experience of pregnancy complication (β = 4.37, ρ = 0.043) influenced PCMC.

**TABLE 7:** Factors associated with perception of person-centred maternity care among post-partum women in Enugu State, Nigeria, 2019.

Factors		B	95% wald Confidence interval		Sig
			Lower	Upper	
Age at marriage	(Intercept)	45.91	41.03	50.80	0.000
	15–19 years	0^a^	—	—	—
	20–29 years	3.46	0.63	6.30	0.017*
	30–49 years	−5.56	−10.27	−0.86	0.020*
Respondent’s occupation	Farmer	0^a^	—	—	—
	Unemployed/student	−4.53	−9.93	0.87	0.100
	Self-employed	−7.50	−12.76	−2.25	0.005*
	Salaried worker (govt)	−2.00	−7.78	3.79	0.498
	Artisan/petty trading	−0.58	−5.47	4.31	0.816
Partner’s occupation	Farmer	0^a^	—	—	—
	Unemployed/student	6.98	−0.71	14.66	0.075
	Self-employed	5.17	−0.50	10.83	0.074
	Salaried worker (govt)	7.12	1.11	13.12	0.020*
	Artisan/petty trading	5.19	−0.08	10.46	0.053
Initiation of ANC	First trimester	0^a^	—	—	—
	Second trimester	−0.86	−3.17	1.46	0.469
	Third trimester	−6.78	−11.25	−2.31	0.003*
	No ANC	−8.41	−19.35	2.53	0.132
Participation in decision making	Low	0^a^	—	—	—
	High	−10.41	−12.69	−8.12	<0.001*
Domestic violence (DV) experience	No DV experience	0^a^	—	—	—
	DV experience	3.60	0.99	6.22	0.007*
Facility type	Public hospital	0^a^	—	—	—
	Public health centre	18.10	13.65	22.55	<0.001*
	Private/mission hospital	4.01	1.39	6.63	0.003*
Provider type	Nurse/midwife	0^a^	—	—	—
	Doctor	2.49	−0.79	5.77	0.137
	CHEW	−10.30	−14.48	−6.13	<0.001*
	Non-skilled attendant	−16.55	−23.56	−9.54	<0.001*
Pregnancy complication (PC)	No PC experience	0^a^	—	—	—
	PC experience	4.37	0.13	8.62	0.043*
(Scale)	—	133.74^b^	117.36	152.40	—

a Set to zero because this parameter is redundant.

b Maximum likelihood estimate.

*Significant at ρ < 0.05

## Discussion

The purpose of this study was to assess the perception of PCMC and its associated factors among post-partum women in Enugu State, Southeast Nigeria. Analysis of the findings reveals three areas that need to be explored further. The first is the psychometric properties of the PCMC scale. The second is the fact that PCMC is generally inadequate. The third relates to the factors associated with PCMC that can be considered for inclusion in intervention strategies to improve PCMC in Enugu State, Nigeria.

We found the PCMC scale to be a valid and reliable instrument for measuring women’s experiences of responsive and respectful care in the study population. The construct validity was high for the 22 items with adequate communalities and high rotated factor loading. The goodness-of-fit indices were generally adequate. The criterion validity was also high because total PCMC scores correlated strongly with women’s satisfaction with quality of maternal health services. Nonetheless, the two-factor solution for our data did not represent clear conceptual domains. For instance, factor 1 was dominant and included 20 items from the three domains. Consistent with evidence from Indian PCMC validation [4], we regrouped the retained items into three conceptual domains to provide the sub-scales for Dignity and respect, Autonomy and communication, and Supportive care. The full PCMC scale and its sub-scales had high reliability. Although our 22-item PCMC scale is shorter than the Kenyan and Indian PCMC scales their psychometric properties are consistent confirming that our PCMC scale has high construct validity, criterion validity, and reliability [1, 4]. Furthermore, our PCMC scale did not retain any factor with low communality and inadequate loading as were the case in previous studies [1, 4]. For instance, despite being theoretically relevant to PCMC, verbal and physical abuse are culturally accepted as normal and helpful to ensure positive childbirth outcomes in Nigeria and are underreported [20, 30]; and might not represent a good measure for women’s experiences with maternity care.

The study found that most women had low to medium scores on full PCMC scale and sub-scales, which are comparable to evidence from previous studies [1, 4–6]. The least proportion of women with high perception was communication and autonomy sub-scale, while highest proportion was respect and dignity dimension. However, there were considerable variations in individual PCMC items. Most women had high perceptions of respectful care, friendly care, visual privacy, and record confidentiality which are similar to existing evidence [4–6]. Lower score on communication and autonomy resulted from limited consented care, inadequate explanation of procedure and medicines, low involvement in decisions about women’s care, and not calling of women by their names. Our findings regarding these items of autonomy and communication were much lower than findings from previous studies [4–6]. Supportive care was constrained by restrictive labour companionship, inadequate support of anxious women, poor control of pain, dirty environment, and inadequate staffing. Evidence on these supportive care variables from previous studies are mixed suggesting that PCMC varies with context [4–6]. Improving PCMC would involve strengthening respectful care, visual privacy, and record confidentiality, informed consent, and interpersonal communication, and addressing gaps in facility-level drivers of low PCMC.

This study revealed that marriage at 20–29 years had a significant positive relationship with women’s perception of PCMC, similar to findings of a previous study [11]. Yet, marriage at 30–49 years had a significant inverse relationship with women’s perception of PCMC. The influence of age at marriage on PCMC might not simply reflect age, but also economic and educational empowerment given that nearly 60% of women in our sample belong to rich quintiles and most women have a minimum of secondary education. In Nigeria, women with no education marry 6 years earlier than women with secondary education, whereas women in the lowest wealth quintile marry eight years earlier than women in the highest quintile [24]. We argue that women, who marry at age 20–29 years, are better empowered, have higher expectation of care and can recognise low-quality care and advocate for improved care. However, women at age 30–49 years are better empowered than at age 20–29, giving them more freedom to take decisions and make personal choices [31], and as such, they tend to have a poorer perception of PCMC.

We found that self-employment had significant, but an inverse relationship with women’s perception of PCMC. An increase in self-employment would result in decrease in PCMC among self-employed women. This finding is comparable with evidence in Kenya which found that employment status predicted women’s perception of PCMC [7]. However, while the study in Kenya dichotomized occupation into unemployed and employed, our study used five occupational categories. Two factors could explain our findings. First self-employment could enhance women’s participation in household decision-making for their own healthcare [32]. Secondly, self-employment increases women’s economic empowerment, which means that women can effectively demand better maternity care [8, 32]. The empowered state makes the women more demanding of better PCMC, and as such, they tend to have a poorer perception than those who are less empowered, who might be more grateful for whatever PCMC they might get. As perception of PCMC varies with socio-economic status [5, 7], an increase in women’s labour participation that promotes self-employment is needed to improve person-centred maternity care.

Marriage to government workers had a significant positive relationship with women’s perception of PCMC. In Kenya, women’s perception of PCMC were associated with marrying petty traders but not government workers [7]. Prior studies indicate that men can provide substantial practical, financial, and emotional support to overcome demand-side barriers to accessing maternal health services and improve positive childbirth experiences [33]. It might be that in this study, government workers cared more for their pregnant partners and provided support during pregnancy and childbirth, which improved their perception of PCMC. Also, high cost is an important barrier to respectful maternity care and skilled delivery service in Nigeria [20]. It might be the case that women who are married to government workers are covered by formal sector health insurance scheme [34], or free maternal healthcare programme since evidence of public sector employment of a partner guaranteed women’s access to free care [35].

High participation in household decision-making was found to have an inverse relationship with women’s perception of PCMC in this study. Our results contrast findings of a prior study in Kenya which found that PCMC was not significantly related to participation in household decision-making [7]. In Nigeria, healthcare decisions for women are mostly made by their husbands/partners without women’s involvement [36]. It might be that low women’s decision-making autonomy limits women’s expectation of quality of maternity care, social power between women and providers, and women’s capacity to demand better care in Nigeria. Conversely, women who participate highly in household decisions are better aware of their rights to person-centred care and tend to have increased self-confidence thereby reducing power differential between health providers and women [20].

This study further revealed that women who had no domestic violence experience had significantly higher perception of PCMC than those who experienced domestic violence. Our finding, which is consistent with evidence from a prior study in Kenya [7], is expected because women who experience gender-based violence are disempowered and more vulnerable to dominance by providers [37]. Women who experience domestic violence are emotionally challenged. Women even when receiving technically sound care but lacking in emotional support perceive it as low-quality care [9]. Also, domestic violence limits women’s decision-making power regarding their reproductive health and have been associated with poor maternal health outcomes [38].

Trimester of commencing antenatal care predicted women’s perception of PCMC in this study. Women who commenced antenatal care during the third trimester were more likely to have a lower perception of person-centred maternity care than women who started antenatal care in their first trimester. Our findings are inconsistent with results of a previous Kenyan study [7]. Failure to initiate antenatal care early is a potential risk for complications during pregnancy and childbirth [39]. Tailored group educational activities and peer support motivates behaviour change among pregnant women and increases women’s satisfaction with maternity care [39]. In this study, late initiation of antenatal care meant that women are not familiar with the health system and might not have the benefit of psychological support and sharing of experiences which help women feel more empowered as decision makers during childbirth [40].

Moreover, women who were delivered in health centres and private/mission hospitals had higher PCMC scores than those delivered in public hospitals. Similar findings of higher PCMC were also found in health centres and private hospitals in Kenya [7]. Our findings support the evidence of higher interpersonal quality of maternal healthcare in health centres and private than public hospitals [9, 13, 15]. Conversely, indices of clinical quality of maternal health were higher in public hospitals than private hospitals and health centres [13, 14]. In this study, higher PCMC scores in health centres and private hospitals may be due to low provider-patient ratio which reduces the strain on provider-patient interaction [7]. Equally, higher PCMC scores in health centres might reflect closer ties between providers and women in closely knitted communities that health centres serve [7] and effect of citizen participation in governance of health centres [41]. In Nigeria, users have better perception of health workers in private facilities because private facilities greatly emphasize interpersonal quality [42].

Type of birth attendant was also found to predict women’s perception of PCMC in this study. PCMC was inversely and significantly related to delivery by community health workers and non-skilled attendants, although we expected a direct relationship given that negative attitudes and behaviours are commonly ascribed to trained professionals especially doctors and nurses [43]. Although women who were delivered by doctors received higher PCMC than those delivered by nurses, delivery by doctors was not significantly predictive. By contrast, PCMC was directly and significantly related to delivery by doctors in Kenya [7]. Higher perception of PCMC among women delivered by doctors than nurses is consistent with a Nigerian study showing that healthcare users have a better perception of doctors than nurses [42]. It could be that negative attitudes and behaviours are more common among nurses than doctors as hostile and impersonal behaviour from nurses and midwives are common reasons for dissatisfaction with quality of maternal health services in South-east Nigeria [44].

Furthermore, our study revealed that women who had pregnancy complications had higher PCMC scores than those without pregnancy complications; and experience of pregnancy complication significantly predicted perception of PCMC. Comparable results were found in Kenya, where women with severe pregnancy complication reported higher PCMC than other women [7]. By contrast, we expected that women with pregnancy complications will have significantly lower PCMC than those without complications. Our expectation is consistent with findings in previous studies showing that there were higher incidents of disrespectful care among women who experience pregnancy complications and longer labour durations requiring instrumental delivery and caesarean birth [11, 16–19]. It could be that survivors of pregnancy complication are more satisfied with their positive pregnancy outcomes and tend to report exaggerated positive patient experiences.

This study builds on current literature by adding validating PCMC scale in a Nigerian population and identifying factors that may be considered for inclusion in intervention strategies to improve PCMC in Nigeria. However, this study could have recall bias, though our respondents seemed to recall their childbirth experiences vividly. While women recall childbirth experiences accurately within twenty years [45], we adopted 9 weeks post-partum following a previous study [1] and because we thought that women would have the best chances of recall in the first few weeks following the post-partum period. Secondly, sampling bias is possible as only women who gave birth to live babies and attended immunization clinics were included. The study, therefore, potentially excluded women with stillbirths and neonatal deaths who may have had negative childbirth experiences. Finally, demographics of women attending immunization clinics in our study sites may not completely reflect demographics of post-partum women in Nigeria, possibly limiting generalizability of the study.

### Conclusion

Evidence from this study indicate that PCMC scale is a valid and reliable instrument for measuring responsive and respectful maternity care. The study also reveal that PCMC is generally inadequate and associated with six patient characteristics (age at marriage, self-employment, married to government worker, high participation in household decisions, domestic violence experience, and initiation of antenatal care in the third trimester); two facility characteristics (facility type and provider type); and service type (pregnancy complication). This information should inform the design of interventions to promote positive childbirth experiences and evaluation of changes in the quality of maternity care.

## Data Availability

The original contributions presented in the study are included in the article/Supplementary Material, further inquiries can be directed to the corresponding author.
